# Development of mesothelin-specific CAR NK-92 cells for the treatment of gastric cancer

**DOI:** 10.7150/ijbs.64630

**Published:** 2021-09-03

**Authors:** Bihui Cao, Manting Liu, Jingjun Huang, Jingwen Zhou, Junping Li, Hui Lian, Wensou Huang, Yongjian Guo, Shuo Yang, Liteng Lin, Mingyue Cai, Cheng Zhi, Jingqiang Wu, Licong Liang, Yuling Hu, Hong Hu, Jinping He, Baoxia Liang, Qi Zhao, Kangshun Zhu

**Affiliations:** 1 Department of Minimally Invasive Interventional Radiology and Department of Radiology, The Second Affiliated Hospital of Guangzhou Medical University, Guangzhou, Guangdong, 510260, China.; 2 MoE Frontiers Science Center for Precision Oncology, Faculty of Health Sciences, University of Macau, Macau SAR, 999078 China.; 3 Department of Pathology, The Second Affiliated Hospital of Guangzhou Medical University, Guangzhou, 510260, China.; 4 Department of Pharmacy, Guangzhou Medical University, 511436, China.

**Keywords:** Gastric cancer, mesothelin, chimeric antigen receptor, NK-92, PDX

## Abstract

**Background:** The application of chimeric antigen receptor (CAR) NK cells in solid tumors is hindered by lack of tumor-specific targets and inefficient CAR NK cell efficacy. It has been reported that mesothelin (MSLN) may be an ideal immunotherapy target for gastric cancer. However, the feasibility of using anti-MSLN CAR NK cells to treat gastric cancer remains to be studied.

**Methods:** MSLN expression in primary human gastric cancer, normal tissues and cell lines were detected. MSLN and CD19 targeted CAR NK-92 (MSLN- and CD19-CAR NK) cells were constructed, purified and verified. N87, MKN-28, AGS and Huh-7 cells expressing the GFP and luciferase genes were transduced. Cell- and patient-derived xenograft (PDX) were established via NSG mice. The ability of MSLN-CAR NK cells to kill MSLN-positive gastric cancer cells were evaluated *in vitro* and *in vivo*.

**Results:** MSLN-CAR NK cells can specifically kill MSLN-positive gastric cancer cells (N87, MKN-28 and AGS), rather than MSLN negative cell (Huh-7), *in vitro*. Moreover, compared with parental NK-92 cells and CD19-CAR NK cells, stronger cytokine secretions were secreted in MSLN-CAR NK cells cocultured with N87, MKN-28 and AGS. Furthermore, MSLN-CAR NK cells can effectively eliminate gastric cancer cells in both subcutaneous and intraperitoneal tumor models. They could also significantly prolong the survival of intraperitoneally tumor-bearing mice. More importantly, the potent antitumor effect and considerable NK cell infiltration were observed in the patient-derived xenograft treated with MSLN-CAR NK cells, which further warranted the therapeutic effects of MSLN-CAR NK cells to treat gastric cancer.

**Conclusion:** These results demonstrate that MSLN-CAR NK cells possess strong antitumor activity and represent a promising therapeutic approach to gastric cancer.

## Introduction

Globally, gastric cancer was the fourth-leading cause of cancer related death in 2020, resulting in approximately 768,793 deaths 1. More likely to be diagnosed in males, gastric cancer rates in men are nearly twice as high as those in women, and they vary widely among different regions and countries, with the highest incidence rates in Eastern and Central Asia and Latin America 1. Although it is steadily declining in incidence and mortality due to prevention strategies, improvement in diet, helicobacter pylori eradication, early detection, standardized surgical techniques, adjuvant or neo-adjuvant chemotherapy, targeted therapy (trastuzumab) and immune checkpoint inhibitors (PD1/PD-L1), 5-year survival rate for advanced gastric cancer remains low across the world 2-4. Thus, exploring novel treatment strategies for gastric cancer is essential and of great importance.

Chimeric antigen receptor (CAR) T cell therapy has shown a tremendous clinical efficacy for tumor treatment, especially the utilization of CD19-CAR T cells in B cell malignancies 5-7. However, to generate a therapeutic dose of autologous CAR T cells is time-consuming and difficult, due to the limited cell division and short life span of autologous T cells 8. What is more, off-target, cytokine release syndrome (CRS), the risk of GVHD, and other adverse reactions, are restricting the further clinical applications of CAR-engineered primary T cells 9. As an alternative to CAR T cell therapy, engineering NK cells to express a CAR that target a specific antigen on target cells has gained much attention. CAR NK cell therapy not only circumvents above challenges but also presents additional major merits. First, genetically modified CAR NK cells can target tumor cells without the concern of GVHD 10. Then, NK cell line or iPSC-NK offer an “off-the-shelf” opportunity to culture unlimited allogeneic NK cells 11. In addition, CAR NK cells eliminate tumors not only through the ability of CAR to specifically recognize antigen-expressing tumors but also through NK cell intrinsic receptors and antibody-dependent cell-mediated cytotoxicity (ADCC) manner 12, thus less likely allowing disease escape via tumor heterogeneity or downregulation of the CAR antigens as shown with CAR T cell therapy 11. Furthermore, allogeneic NK cells have been proved to be safe after infusion for adoptive immunotherapy in cancer patients. These above capability, together with the HLA-mismatching requirements between CAR NK cells and the recipient, may pave the way for a truly off-the-shelf possibility that might benefit many more candidates 13.

Unlike CD19 in hematological malignancies, there are few tumor-specific antigens that are widely used to therapeutically target solid tumors since most of them are also expressed on critical tissues. Mesothelin (MSLN) is a tumor-differentiation antigen. It is a cell-surface glycoprotein with normal expression restricted to mesothelial cells lining the pleura, peritoneum, and pericardium. However, it is also highly overexpressed in many cancers, including malignant mesothelioma, pancreatic cancer, ovarian cancer, lung adenocarcinoma, endometrial cancer, biliary cancer, pediatric acute myeloid leukemia, as well as gastric cancer 14. Because MSLN is expressed only on non-critical tissues, the risk of off-target toxicity is decreased. Multiple studies have validated MSLN as an attractive target for CAR T or NK immunotherapy in solid tumors 14, 15; however, using CAR NK cells to target MSLN against gastric cancer has never been explored. In this study, we established MSLN-specific CAR NK-92 (MSLN-CAR NK) cells and explored their antitumor activities on gastric cancer models *in vitro* and *in vivo*.

## Materials and methods

### Immunohistochemistry assay

To detect MSLN expression in gastric cancer and normal gastric tissues, we performed immunohistochemistry (IHC) assay. All the human samples collection were approved by the Institutional Review Boards of the Second Affiliated Hospital of Guangzhou Medical University. Different levels of MSLN expression in 75 cases gastric cancer and 20 cases gastric normal paraffin-embedded samples (detailed in Supplementary Table 1), were assessed by one experienced pathologist using a 4-point scale. Score 0 means no MSLN expression; scores of 1+, 2++, and 3+++ mean weak to strong expression of MSLN. The percentages of MSLN-positive staining with different scores were also recorded. And following is the detail of IHC procedure:

The samples were fixed with 10% formalin and embedded in paraffin. Sectioned slices were deparaffinized in xylene, rehydrated in graded alcohol, and placed in Tris-buffered saline (TBS) for 15 min. The sections were then submitted to antigen retrieval (sodium citrate buffer, pH 6) and inactivation of endogenous peroxidase. Nonspecific sites were blocked with animal nonimmune serum (Maxvision) and incubated with diluted (1:200) rabbit anti-human MSLN primary antibody (Cell Signaling Technology, clone D9R5G) overnight at 4 °C. After incubation with the corresponding secondary antibody (Maxvision) for 15 min at room temperature, the sections were stained using a detection kit (Maxvision). Cell nuclei were stained with hematoxylin (Sigma). Finally, sections were dehydrated with absolute ethanol. Images of the samples were captured with an Olympus TH4-200 microscope.

### Cell lines

Human gastric cancer cell lines N87, MKN-28, AGS and liver cancer cell line Huh-7, HEK-293T and NK-92 cell lines were purchased from the American Type Culture Collection (Manassas, VA, USA). Cells were cultured in a humidified atmosphere of 5% CO_2_ at 37 °C. Human NK-92 cells were maintained and irradiated with 10 Gy (Biobeam 2000 device, Germany) according to previously described 16. Dulbecco's modified Eagle medium (Gibco) or RPMI-1640 (Gibco) with 10% FBS (Gibco), 100 IU/mL of penicillin (Gibco), and 100 IU/mL of streptomycin (Gibco), were used to culture all other cell lines. N87, MKN-28, AGS and Huh-7 cells were lentivirally transduced with LV5-LUC-GFP-Puro virus (Genepharma, Shanghai, China) expressing the GFP and luciferase (GL) gene. All cells were routinely tested for mycoplasma contamination.

### Lentivirus production

To generate CAR-targeting molecules, an anti-MSLN scFv 17 and anti-CD19 scFv, a NKG2D transmembrane region, combined with 2B4-CD3ζ intracellular signaling domains, were synthesized and cloned into the lentiviral vector pWPXLd-2A-eGFP by Genscript Co., Ltd. (Nanjing, China). Lentivirus particles were produced in HEK-293T cells after polyethyleneimine (Sigma-Aldrich) transfection. The pWPXLd-based lentiviral plasmid, together with two auxiliary packaging plasmids, psPAX2 and pMD.2G, were cotransduced into HEK-293T cells. Lentivirus-containing supernatants were harvested at 48 and 72 hours post-transfection and filtered through a 0.45-µm filter (Milliopore) to remove cell debris.

### Quantitative real-time polymerase chain reaction (qPCR)

The expression of different CAR and cytokine mRNAs was detected via quantitative real-time polymerase chain reaction (qPCR), as previously described. The glyceraldehyde-3-phosphate dehydrogenase (GAPDH) gene was utilized as an endogenous control. The primers for different used CARs were:

- GAPDH - forward: 5'- GCACCGTCAAGGCTGAGAAC -3';
- GAPDH - reverse: 5'- TGGTGAAGACGCCAGTGGA -3';
- scFv of MSLN - forward: 5'- TTATTACTCCTTACAATGGTGCTT -3';
- scFv of MSLN - reverse: 5'- TAGGCTGTGCTGGATGACTT -3';
- scFv of CD19 - forward: 5'- ACTACATCCTCCCTGTCTGCC - 3';
- scFv of CD19 - reverse: 5'- CCACTGCCACTGAACCTTGA - 3'.

### Flow cytometry

To evaluate CAR or GL expression in transduced cells, flow cytometry detections for CD19-, MSLN-CAR NK-92 cells, along with N87 GL, MKN-28 GL, AGS GL, and Huh-7 GL, were performed on a NovoCyteTM (ACEA Biosciences). Transduced CAR NK or GL cells were sorted by a FACS sorter (Beckman Coulter MoFlo XDP, USA). All the data was analyzed by FlowJo software (FlowJo).

### *In vitro* cytotoxicity and cytokine release assay

The N87 GL, MKN-28 GL, AGS GL, and Huh-7 GL cells were incubated with parental NK-92, CD19- or MSLN-CAR NK-92 cells at the indicated effector to target (E:T) ratios of 16:1, 8:1, 4:1 and 2:1 in triplicate wells of U-bottomed 96-well plates at 37 °C. Target cell viability was detected 6 hrs later by adding 100 µL/well substrate D-Luciferin (potassium salt; Cayman Chemical) resolved at 150 µg/mL. The viability percentage (%) was calculated as: experimental signal/maximal signal, and the specific cell lysis was computed using a formula: 100%-viability percentage.

Target cells (1×10^4^) were incubated with effector cells (2×10^4^) in 96-well U-bottomed plates for 24 hrs. The culture supernatants were then collected and analyzed for the secretion of interferon-γ (IFN-γ), Granzyme B, granulocyte-macrophage colony-stimulating factor (GM-CSF) and perforin via enzyme-linked immunosorbent assay (ELISA) kits (R&D Systems) according to the instructions.

### Cell derived xenograft (CDX) models for *in vivo* treatment

Six- to eight-week-old female NSG (NOD-Prkdc^scid^IL2rg^tm1^/Bcgen, BIOCYTOGEN, Beijing, China) mice were raised under specific pathogen-free conditions and were provided autoclaved food and water at the Experimental Animal Center of Guangzhou Medical University (Guangzhou, China). All animal experiments were performed according to the applicable guidelines and regulations approved by Guangzhou Medical University Experimental Animal Care Commission.

For the cell line-derived gastric cancer subcutaneous xenograft models, 2×10^6^ N87 cells in 100 μL PBS were subcutaneously inoculated into the right flanks of NSG mice (on day 0). When tumor volume reached approximately 50 mm^3^, the mice were randomly divided into three groups: NC, CD19-CAR NK and MSLN-CAR NK (n=5), and received PBS or 5×10^6^ irradiated CAR NK (MSLN- or CD19-CAR NK) cells intravenously (every 7 days, 3 times in total). Tumor volume was measured regularly with a caliper and calculated with the following equation: tumor volume=(length×width^2^)/2. On day 32 after tumor inoculation, all mice were sacrificed and analyzed. To detect cytokine level in mice model, plasma was harvested from the peripheral blood of mice by centrifugation for 5 min at 300 ×g at room temperature. Supernatant was transferred into fresh tubes and centrifuged again for 5 min at 2,000 ×g. Plasma was stored at 80 °C or detected immediately via enzyme-linked immunosorbent assay (ELISA) kits (R&D Systems) according to the instructions.

In order to construct intraperitoneal gastric cancer models, 1 × 10^6^ MKN-28 GL cells in 100 μL PBS were injected into the peritoneal cavity of NSG mice on day 0, to mimic the carcinoma *in situ*. Ten days after tumor cells injection, the mice were subjected to bioluminescence imaging (BLI) and randomly divided into three groups (n=5): NC, CD19-CAR NK, and MSLN-CAR NK. Mice were administrated with PBS (NC), or 5 × 10^6^ irradiated MSLN- or CD19-CAR NK cells suspended in 100 μL PBS intraperitoneally on day 10, 15, 20 and 25. Bioluminescence imaging (BLI) was performed and quantified frequently using the IVIS system (IVIS, USA) following the previously described protocol. Mice were killed when their body weight loss was greater than 20% of the initial weight, they were unable to ambulate, or their tumor became ulcerated in the control groups.

### Patient derived xenograft (PDX) models for CAR NK-92 cell treatment

For MSLN-CAR NK efficacy studies using gastric tumor patient-derived xenograft (PDX) models, tumor-bearing mice with a tumor size about 50 mm^3^ were randomly divided into (n=5): (a)NC; (b) CD19-CAR NK cells and (c) MSLN-CAR NK cells. A dose of 5×10^6^ irradiated effector cells were intravenously injected via the tail vein in 100 μL PBS every 7 days. Tumor volume was measured regularly. NK cell infiltration in tumor was tested by flow cytometry and IHC. At the same time, all important mouse organs, including heart, liver, spleen, lung and kidney were harvested, fixed with 4% paraformaldehyde and stained with hematoxylin-eosin (H&E).

### Western Blotting analysis

For Western blotting (WB), gastric cancer or tissues were washed with PBS and directly lysed by ultrasonic cell disrupter (Sonics) and Laemmli buffer (Bio-Rad Laboratories, USA). Lysates were electrophoretically separated on an 8% or 10% gradient SDS-PAGE gel (Bio-Rad Laboratories) and transferred to a nitrocellulose membrane. Subsequent procedures were modified from the standard protocol. Membranes were probed with rabbit anti-human MSLN primary antibody (Cell Signaling Technology, clone D9R5G) and anti-human CD3 (Abcam, clone SP7) primary antibodies.

### Statistics

Data are shown as mean ± SD or SEM. Differences between groups were analyzed by one way ANOVA with Bonferroni post-tests. The Kruskal-Wallis test was used to compare the non-normally distributed endpoints. For survival data, Kaplan-Meier curves were plotted and compared by log-rank test. GraphPad Prism 8.0 was utilized for the statistical calculations. *p < 0.05, **p < 0.01, and ***p < 0.001 were considered statistically significant.

## Results

### Expression profile of MSLN in human normal, gastric cancer tissues and cell lines

**Figure 1:**
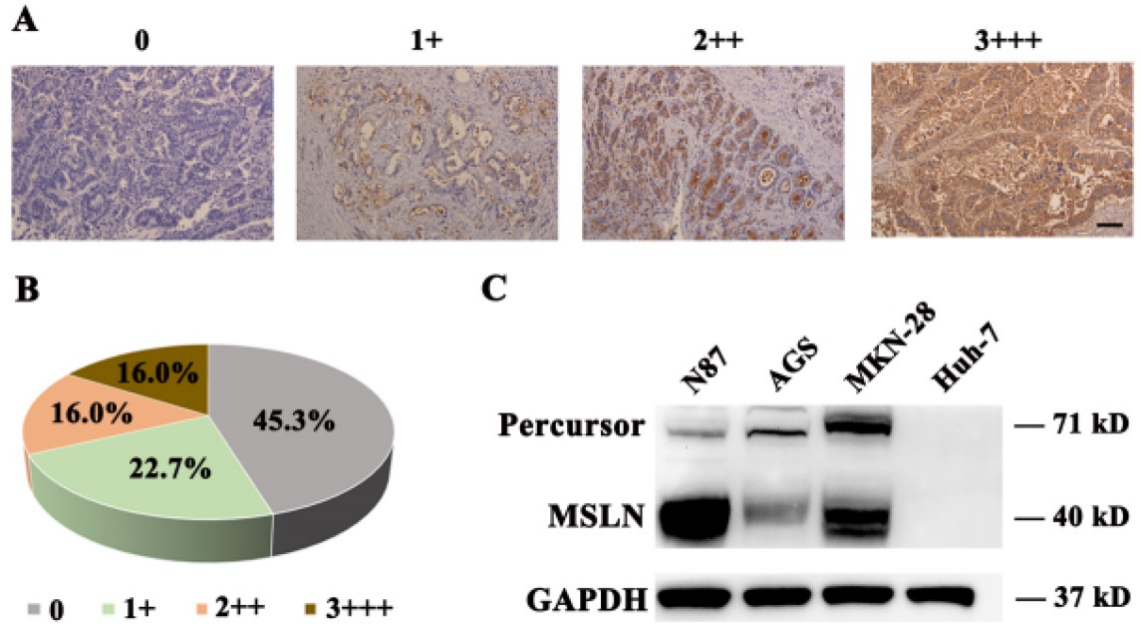
** MSLN expression in gastric cancer and cell lines. A**. Different levels of MSLN expression were evaluated using a 4-point scale at 200×magnification, scale bar: 100 µm. **B**. The percentages of different MSLN expression in 75 primary gastric cancer samples. **C**. Detection of MSLN expression in N87, MKN-28, AGS and Huh-7 cells, by Western Blot.

To detect MSLN expression in gastric cancer tissue, we performed immunohistochemical staining for MSLN in 75 primary gastric cancer and 20 normal gastric samples and found robust expression in 54.7% (41/75) of gastric cancer samples (Fig. 1A & B), but negatively found in normal gastric tissue (data not shown). Then we evaluated MSLN expression in three human gastric cancer cell lines, including N87, MKN-28, and AGS, and one liver cancer cell line, Huh-7, by immunoblotting analysis. MSLN expressed positively in N87, MKN-28, and AGS cell lines, but negatively in Huh-7 (Fig. 1C). Collectively, these results demonstrate that MSLN expression was upregulated in both gastric cancer primary samples and cell lines.

### Generation of CAR NK cells

**Figure 2:**
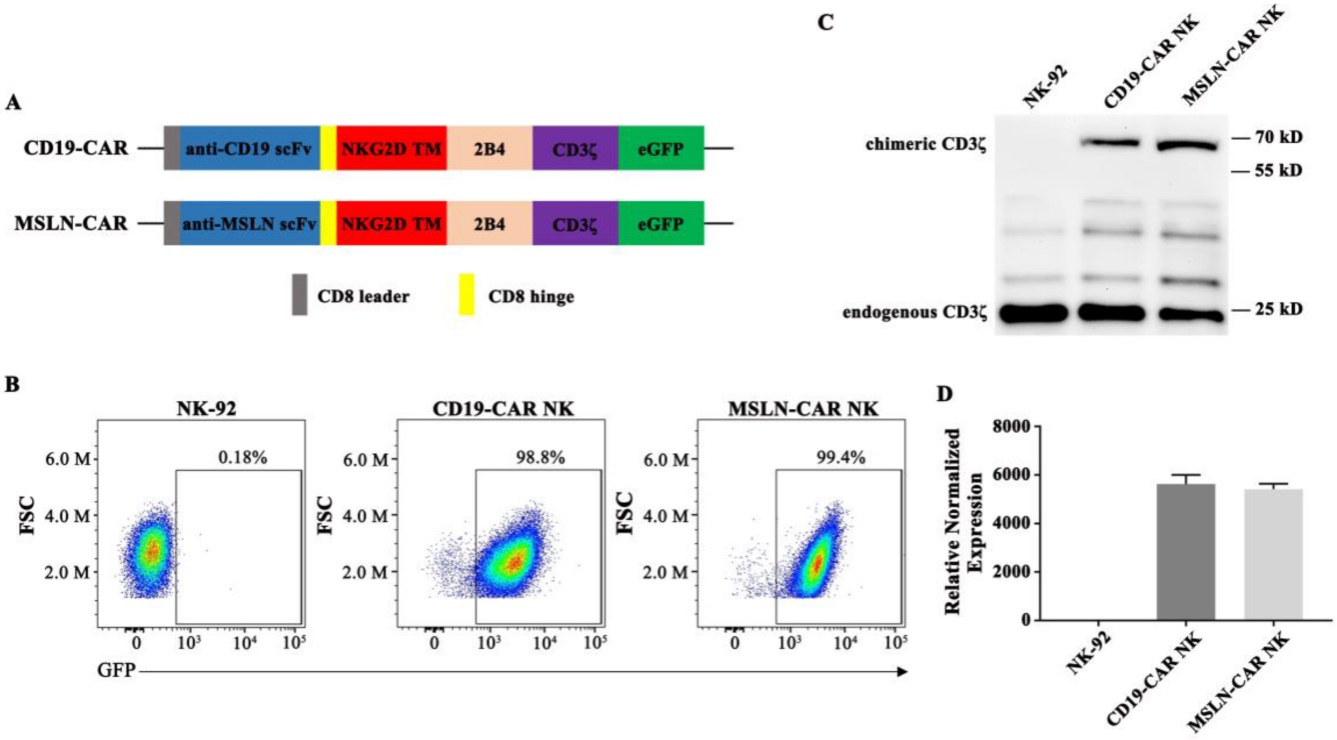
** Generation of MSLN- and CD19-CAR NK cells. A**. Schematic representation of MSLN- and CD19-CAR molecules. **B**. Percentage of MSLN- and CD19-CAR transduced CAR NK cells detected by flow cytometry. GFP served as a marker of CAR expression. **C**. Western blot analysis of CAR expression in transduced MSLN- and CD19-CAR NK cells. A CD3ζ-specific antibody was used to detect endogenous and chimeric CD3ζ. **D**. Relative expressions of different CAR mRNAs normalized to GAPDH in CAR NK cells were detected by qPCR.

The structures of CAR molecules are shown in Fig. 2A. The MSLN- or CD19-CAR molecule including the MSLN or CD19 scFv, a CD8 hinge, a NKG2D transmembrane region, followed by the intracellular domains of co-stimulatory 2B4 and CD3ζ. NK-92 cells were transduced with the MSLN- or CD19-specific CAR vector to generate MSLN- or CD19-CAR NK cells (Fig. 2A). For enrichment of MSLN- and CD19-CAR NK cells, two or three rounds of cell sorting using a FACS sorter were carried out. Positive cells were enriched based on the expression of green fluorescence protein (GFP) marker. Flow cytometry results indicated that CAR molecules were expressed and highly purified in CAR NK cells with GFP reporter (99.4% and 98.8%, respectively) (Fig. 2B) and PE-labelled MSN protein (Fig. S2). Western Blot and qPCR results further confirmed that the CAR sequences were introduced and expressed in NK-92 cells (Fig. 2C & D). These results verified that CAR NK-92 cells were generated successfully and could be used as effector cells.

### MSLN-CAR NK cells specifically targeted MSLN-positive cancer cell lines *in vitro*

**Figure 3:**
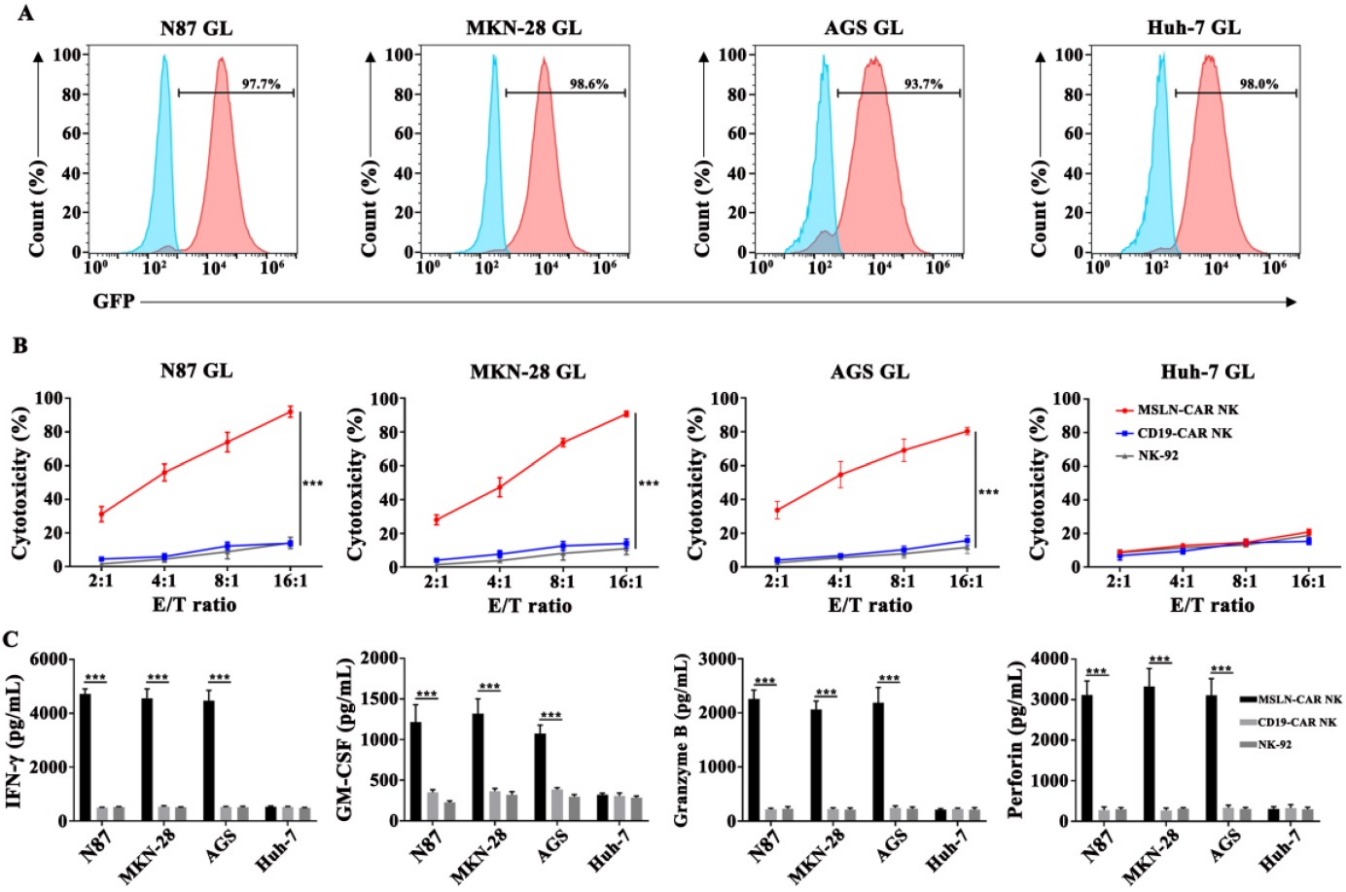
** MSLN-CAR NK cells showed strong antitumor activity against MSLN-positive gastric cancer cell lines *in vitro*. A**. GL expression was detected in N87 GL, MKN-28 GL, AGS GL and Huh-7 GL cell lines by flow cytometry. GFP served as a marker of luciferase expression. **B**. The effector cells were co-cultured for 6 hrs with target cells (1×10^4^ ) at E:T ratios of 2:1, 4:1, 8:1 and 16:1 in a total volume of 100 µL. **C**. Detection of IFN-γ, GM-CSF, Granzyme B and perforin secretion by effector cells after coculture with target cells for 24 hrs at an E:T ratio of 2:1. Data reflected the mean ± SEM of three independent experiments. *p < 0.05, **p < 0.01, and ***p < 0.001.

To examine the cytotoxicity of CAR NK cells against gastric cancer cell lines *in vitro*, a LV5-LUC-GFP-Puro transgene was lentivirally transduced into cancer cell lines and thereby constructed the N87 GL, MKN-28 GL, AGS GL and Huh-7 GL cell lines (Fig. 3A, Fig. S3). We then performed a 6-hr killing assay of MSLN- and CD19-CAR NK cells for GL expressed cells. The results showed that MSLN-CAR NK cells exhibited stronger cytotoxicity than CD19-CAR NK or parental NK-92 cells after coculture with N87 GL, MKN-28 GL, AGS GL cell lines at the indicated E:T ratios, especially at high E:T ratios, *in vitro*. While for MSLN negative cells, Huh-7 GL, cytotoxicity efficacy remained low level in MSLN-CAR NK, CD19-CAR NK and parental NK-92 cells (Fig. 3B). In the cytokine secreting assay, we found that CAR-mediated killing was accompanied by high levels of IFN-γ, GM-CSF, Granzyme B and perforin secretion examined via ELISA in coculture supernatants from MSLN-CAR NK cells in the presence of N87 GL, MKN-28 GL and AGS GL (MSLN positive). Cytokine secretion patterns were consistent with the observation in qPCR (Fig. S4). This phenomenon was not observed in CD19-CAR NK or parental NK-92 cells (Fig. 3C). These results revealed that MSLN-CAR NK cells had intrinsic target-dependent cytotoxic activity *in vitro*.

### MSLN-CAR NK cells showed strong antitumor activity against subcutaneous gastric cancer model

**Figure 4:**
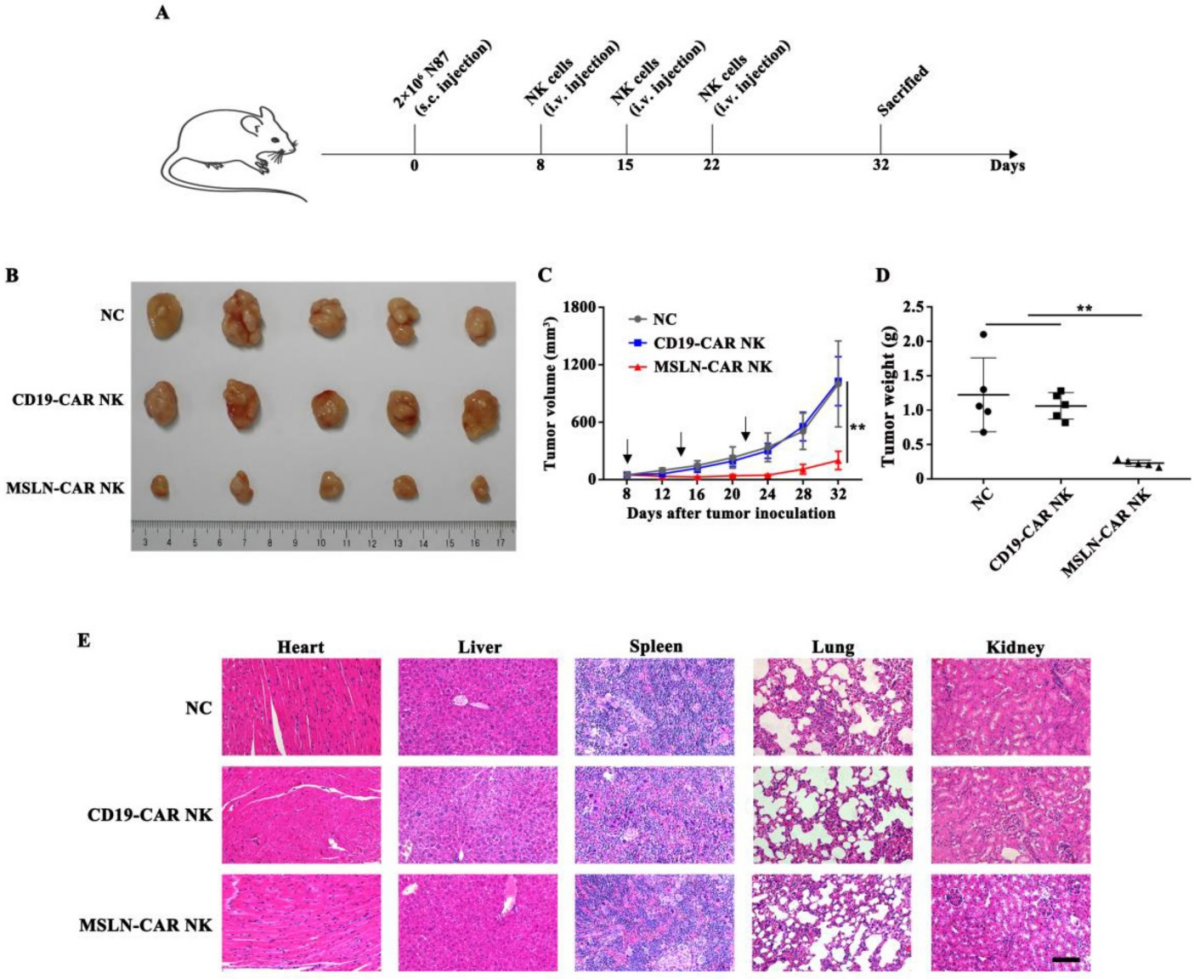
** MSLN-CAR NK cells showed antitumor activity in subcutaneous gastric cancer mouse model. A**. Schematic representation of the experiment. NSG mice received a subcutaneous injection of 2×10^6^ N87 cells. When tumor volume reached about 50 mm^3^, 5×10^6^ CAR NK cells were injected every seven days through the tail vein and tumor volume was measured every four days. **B**. Tumors dissected from different groups at the end point. **C**. Tumor volume curves of N87 subcutaneously injected mice. **D**. Tumor weight in each group at the end point. **E**. Histopathological analysis of murine organ tissues by hematoxylin and eosin (H&E) staining. Representative staining image fields (magnification × 200) are shown. Scale bar represented 100 µm. Error bars denoted the SD. *P<0.05, **P<0.01, ***P<0.001.

Subcutaneous model was inoculated N87 cells on day 0 and intravenously treated with MSLN- or CD19-CAR NK cells on day 8, 15, 22 (Fig. 4A). As shown in Fig. 4B & C, in MSLN-CAR NK group, the calculated tumor volumes sustained relatively stable and at a low level, whereas in NC and CD19-CAR NK groups, tumor volume increased dramatically when compared to the baseline (50 mm^3^). Accordingly, the measured average tumor weight in MSLN-CAR NK group was 0.23 g, which was significantly lighter than NC (1.22 g) and CD19-CAR NK (1.06 g) group (Fig. 4D). Moreover, no obvious damage could be observed in the important organs from the mice treated with CAR NK cells (Fig. 4E). Finally, CAR-mediated *in vivo* killing was accompanied by high levels of IFN-γ; wheras IL-6, a primary reason for cytokine release syndrome, was not detected in subcutaneous mice model (Fig. S1), which indicated a good safety assurance. These results indicated that MSLN-CAR NK cells possessed great potential to eliminate gastric cancer in the subcutaneous model.

### MSLN-CAR NK cells showed strong antitumor activity against intraperitoneal gastric cancer model

**Figure 5:**
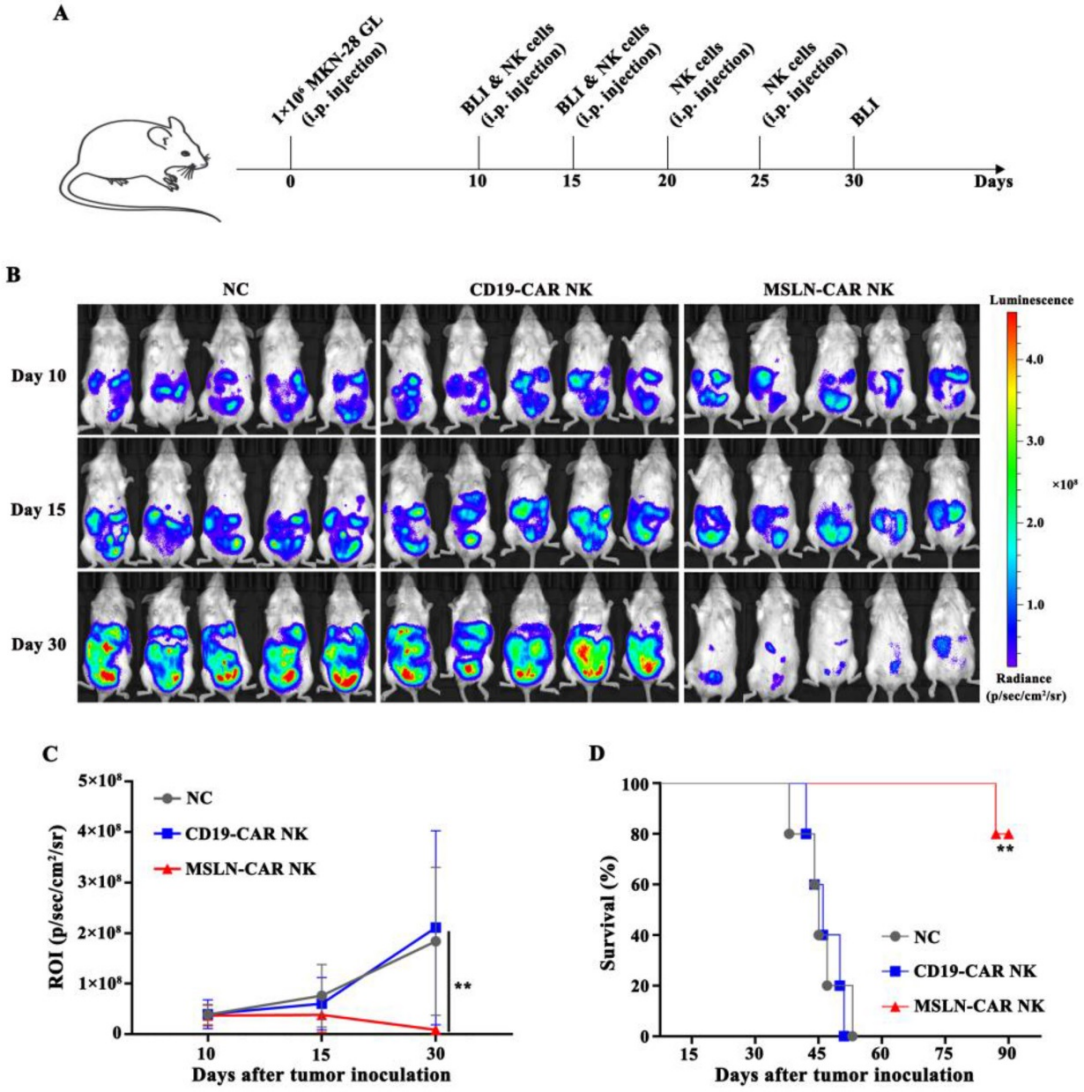
** MSLN-CAR NK cells showed strong antitumor activity *in vivo* in an i.p. gastric cancer model. A**, Schematic representation of the experiment. **B**, BLI of MKN-28 GL intraperitoneally injected mice treated with CAR NK cells (i.p.). On day 0, NSG mice received an i.p. injection of 1 × 10^6^ MKN-28 GL cells. After 10 days, 5×10^6^ CAR NK cells were routinely injected intraperitoneally at the indicated time. On day 10, 15, and 30, BLI was performed. **C**, Statistical analysis of the ROI of each BLI at each time point. Error bars denote the SD, and the results were compared with two-way ANOVA test. **D**, Survival curve of MKN-28 GL intraperitoneally injected mice. The results were performed with Log-rank (Mantel-Cox) test. *P<0.05, **P<0.01, ***P<0.001.

Intraperitoneal gastric cancer model was injected with MKN-28 GL cells on day 0. On day 10, the mice were subjected to BLI, and robust intraperitoneal expansion of tumor cells was observed (Fig. 5A). MSLN-CAR NK cells induced significant regression of MKN-28 GL gastric cancer cells, while tumors in the NC and CD19-CAR NK groups continued to progress, as detected by BLI on day 15 and day 30 (Fig. 4B). Mice in the NC or CD19-CAR NK group, injected with PBS or CD19-CAR NK cells, showed significant enhancement in bioluminescence signals in the abdominal cavity on day 15 and day 30 compared with those on day 10. Wheras the intraperitoneal bioluminescence signals of mice receiving MSLN-CAR NK cells injection significantly decreased on day 15 and 30 (Fig. 4C). In addition, the survival time of mice in MSLN-CAR NK group was significantly longer than that in NC and CD19-CAR NK groups (Fig. 4D). Altogether, these results suggested that MSLN-CAR NK cells could effectively eradicate cell line derived intraperitoneal gastric cancer cells *in vivo*, and prolong the survival time of the tumor-bearing mice.

### MSLN-CAR NK cells induced tumor regression in PDX models *in vivo*

**Figure 6:**
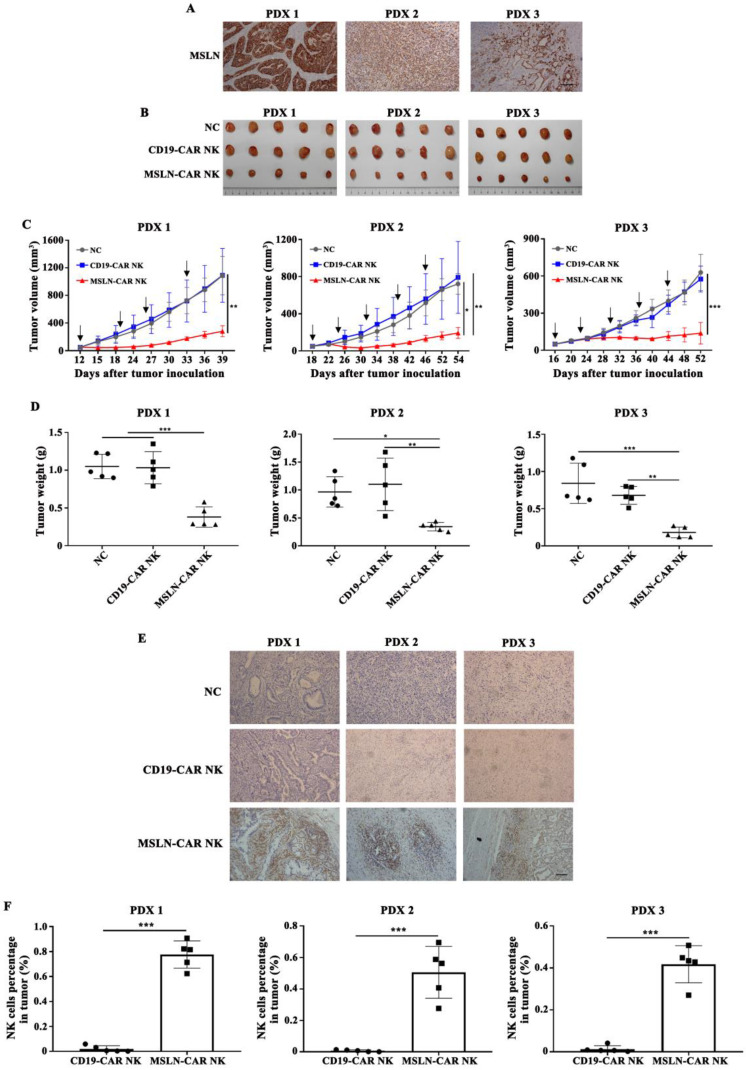
** MSLN-CAR NK cells efficiently inhibit growth of patient-derived xenografts (PDXs) of gastric cancer. A**. MSLN expression in PDX1, PDX2, and PDX3 by IHC (200×), scale bar: 100 µm. **B.** Tumor volume curves after regularly administration with CAR NK cells or PBS (black arrow). **C-D**. At the end of the experiment, the tumors treated with MSLN-CAR NK cells were significantly smaller and lighter than those in the NC and CD19-CAR NK groups. **E**. Representative tumor sections stained with CD56 were shown. The specimens were harvested from PDXs sacrificed after the study was terminated. Nuclei are stained with hematoxylin (200×), scale bar: 100 µm. **F**. Infiltration percentages of MSLN- or CD19-CAR NK cells in tumors were detected by flow cytometry. Data were presented as the mean ± SD. *p < 0.05, **p < 0.01, and ***p < 0.001.

PDX models preserve the heterogeneous pathological and genetic features of the original patient tumors and may offer a precision preclinical model for immunotherapy evaluation. Our results showed that MSLN protein was highly expressed in xenografts of gastric cancer (Fig. 6A), so we tested the effect of MSLN-CAR NK cells in these PDX models. In all three individual PDX models, 5 × 10^6^ CAR NK cells were given by intravenous injection regularly after the tumor volume reached about 50 mm^3^ (Fig. 6B). The potent antitumor effect was observed in the xenografts treated with MSLN-CAR NK cells compared to the CD19-CAR NK cells (Fig. 6B-D). Moreover, NK cell infiltration in MSLN-CAR NK groups were higher than that in CD19-CAR NK groups, which further warranted the therapeutic effects of MSLN-CAR NK cells to treat gastric cancer (Fig. 6E,F). Taken together, our results demonstrated that MSLN-CAR NK cells were able to efficiently suppress the growth of MSLN-positive gastric cancer PDX *in vivo*.

## Discussion

Recently, the understanding of NK cell characteristics has paved the way for novel antitumor therapies 18. NK cells play considerable roles in host innate immunity with high antitumor, antiviral, and antimicrobial activity via two major cytotoxic mechanisms, granulocyte apoptosis mediated by perforin and granzyme as well as antibody-dependent cell-mediated cytotoxicity (ADCC) 19, 20. These cells response much faster than T cells upon stimulation, as they do not require previous sensitization, antibody binding, or pathogen presentation 19. Amounts of clinical settings show that NK cell adoptive immunotherapy (especially expanded NK cells) is a safe, well-tolerated and valuable method, which can enhance NK cell cytotoxicity to treat gastric cancer 21. However, tumors escape from immunological surveillance and the clinical activity of NK cells is modest at best 22, thereby limiting their current therapeutic use. Therefore, genetic manipulation with CAR that recognize a specific antigen uniquely expressed or overexpressed by target cells is proposed as a strategy for reprogramming NK cells to enhance their anti-tumor efficacy 23.

In the present study, we demonstrated potent and specific activity of MSLN-CAR NK-92 cells against MSLN-positive gastric cancer both *in vitro* and *in vivo*. The tumor-associated cell surface antigen MSLN is an important therapeutic target, and MSLN overexpression has been reported in gastric cancer 14. Moreover, overexpression of MSLN plays a central role in cancer cell proliferation, invasion and metastasis via activating PI3K, ERK, and MAPK signaling pathways 14, 24. In addition, in our immunohistochemical analysis, MSLN was positively detected in 54.7% of 75 human primary gastric cancer samples, whereas absent in normal gastric tissue (data not shown), which indicated MSLN might be an ideal target for gastric cancer. Furthermore, MSLN-CAR NK-92 cells induced robust killing efficacy in MSLN-positive cells in xenografts without obvious organ damage. More importantly, IL-6, a primary reason for cytokine release syndrome, was not detected in subcutaneous mice model, this is consistent to the previous study that CAR NK cells do not secret IL-6, a primary reason for cytokine release syndrome when CAR T cells applied in clinical conditions 16. All evidence strongly verified that MSLN-CAR NK-92 cells represented an appropriate and safe treatment method against gastric cancer.

The PDX tumor modelmaintains the heterogeneity of primary tumors and is an excellent model for cancer research as it directly transfers tumors from patients to NSG or NOG mice 25. It has been frequently used to test drug efficacies and identify biomarkers in a plenty of cancers, such as liver, pancreatic, breast, ovarian and prostate cancers 26. Previous studies have revealed that tumors in PDX models are biologically stable and precisely reflect the histopathology, gene expression, genetic mutations, and therapeutic efficacy of the patient tumor 25. Several recent preclinical studies have demonstrated the efficient activity of CAR T cells against hepatocellular carcinoma and gastric cancer 26, 27. However, the application of CAR NK cells in PDX tumor models, including gastric cancer, are still limited. In this study, we established three MSLN-positive gastric cancer PDX models through NSG mice. Interestingly, MSLN-CAR NK cells could lead to complete response in the PDX tumor model, indicating their potent tumor elimination capability. Notably, we observed that the MSLN-CAR NK cells could efficiently infiltrate the tumor tissues from the PDX tumor models, which mimic the microenvironment of the primary tumor to some degree. These warranted the therapeutic effect of MSLN-CAR NK cells to treat gastric cancer and gained potential translational value of MSLN-CAR NK cells in solid tumor.

NK-92 cell line might be suitable in clinical practice, as it can be obtained from molecularly and functionally well-characterized single cell clones under GMP-compliant procedures. NK-92 cells possess several crucial characteristics that make them advantageous over naturally occurring NK cells^20^. They can be favorably expanded *ex vivo* in the presence of IL-2 and display a phenotype similar to activated NK cells, do not show any variation in subtype or phenotypic characteristics, which is a concern when utilizing allogeneic and autologous NK cells. Then NK-92 cell line offers an “off-the-shelf” chance to culture infinite allogeneic NK cells and reduces sensitivity to repeated freeze/thaw processes 11, 28. Furthermore, they lack most KIR receptors but retain armed with a series of activating receptors 29. This increases their cytotoxic potential in the majority of tumor cell lines and possess the capability to conquer tumor heterogeneity *in vitro* and *in vivo*. Most importantly, our previous preclinical study observed potent antitumor activity of CAR NK-92 cells targeting MSLN or B7-H3 in ovarian and non-small-cell lung cancer 30, 31. To date, NK-92 cells have entered FDA approved clinical trials, and encouraging data from recent clinical trials have renewed interest in the field of CAR-engineered NK-92 cancer immunotherapy 22. Consequently, NK-92 cells are suitable and promising for development of CAR-engineered NK cells in clinical application. Meanwhile, we are initiating a phase I clinical study to test the safety, efficacy and translational value of MSLN-CAR NK cells in advanced gastric cancer patients.

Nevertheless, our study is not without limitations. It is less persuasive to use Huh-7, a hepatocellular carcinoma cell line, rather than gastric cancer cell line, as MSLN negative control. We did not use orthotopic gastric cancer model to explore the antitumor activity of CAR NK cells *in vivo*. The cases of gastric cancer and normal samples for IHC are limited since it is difficult to collect these tissues, and individualization screen is needed in potential clinical situations. Further clinical trials are essential to characterize and engineer the potential of NK cells.

## Conclusions

In summary, we have demonstrated the feasibility and efficacy of MSLN-CAR NK cells against gastric cancer *in vitro* and *in vivo*, especially for three MSLN-positive PDX gastric tumor model. Our results suggested that MSLN-targeted NK cell therapy represent a clinically appealing treatment strategy for gastric cancer patients with positive MSLN expression, thus providing the basis for additional investigations in the clinical application of immunotherapy against gastric cancer.

## Supplementary Material

Supplementary figures and table.

## Abbreviations

MSLN: mesothelin
NK: natural killer
CAR: chimeric antigen receptor
GVHD: graft-versus-host disease
GFP: green fluorescent protein
IFN-γ: interferon-γ
CDX: cell derived xenograft
PDX: patient derived xenograft
ADCC: antibody-dependent cell-mediated cytotoxicity
GM-CSF: granulocyte-macrophage colony-stimulating factor
BLI: bioluminescence imaging
IHC: immunohistochemistry
GMP: Good Manufacture Practice
