# Supplementary material for: Development of mesothelin-specific CAR NK-92 cells for the treatment of gastric cancer
Source: Int J Biol Sci. 2021 Sep 3;17(14):3850–61. doi: 10.7150/ijbs.64630 (PMC8495380; doi:10.7150/ijbs.64630)
Supplement: Supplementary file 1 — Supplementary figures and table. [file ijbsv17p3850s1.pdf]

Supplementary file for

Development of mesothelin-specific CAR NK-92 cells for the treatment of gastric cancer

Table S1. Histologic diagnoses and clinical characteristics of patients.

	Years mean±SD	CEA (μg/L) mean±SD	CA72-4(U/mL) mean±SD	Sample number
Normal	55 ± 20.6	2.9 ± 0.93	4.2 ± 1.4	20
Gastric Cancer	56 ± 15.9	47 ± 72.8	34 ± 58.6	75
Histotype				
Tubular				15
Papillary				7
Poorly differentiated				30
Mucinous				14
Signet ring carcinoma				9

SD: standard deviation.

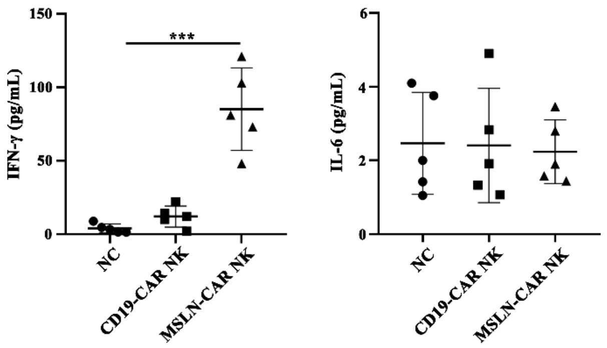

Figure S1. Cytokine levels in plasma of individual mice obtained from blood at 2 weeks after CAR NK cells injection in subcutaneous tumor model. Data reflected the mean ± SEM of three independent experiments. *p < 0.05, **p < 0.01, and ***p < 0.001.

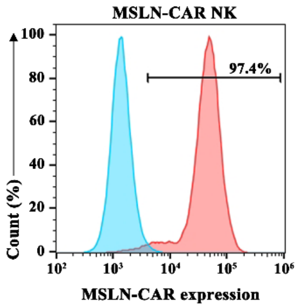

Figure S2. CAR expression by MSLN-CAR NK-92 cells was determined by flow cytometry with PE-Labeled Human Mesothelin Protein (ACROBiosystems, red area). Parental NK-92 cells served as control (blue area).

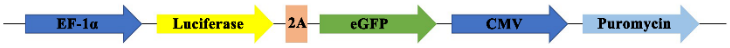

Figure S3. Diagram of LV5-LUC-GFP-Puro (GL) plasmid.

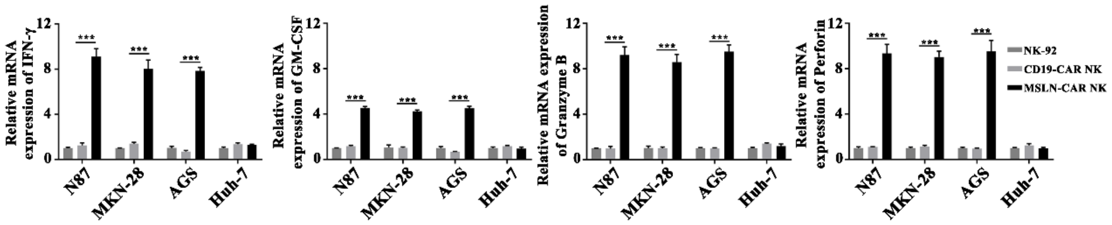

Figure S4. Relative mRNAs expressions of IFN-γ, GM-CSF, Granzyme B and perforin normalized to GAPDH in CAR NK cells were detected by qPCR.
